# Clinic accessibility and clinic-level predictors of the geographic variation in 2009 pandemic influenza vaccine coverage in Montreal, Canada

**DOI:** 10.1111/irv.12227

**Published:** 2014-01-02

**Authors:** Katia M Charland, Luc de Montigny, John S Brownstein, David L Buckeridge

**Affiliations:** a Surveillance Lab, McGill Clinical and Health Informatics, McGill UniversityMontreal, QC, Canada; b Department of Epidemiology, Biostatistics and Occupational Health, McGill UniversityMontreal, QC, Canada; c Children's Hospital Informatics Program, Children's Hospital BostonBoston, MA, USA; d Division of General Pediatrics, Department of Medicine, Children's Hospital Boston, Harvard Medical SchoolBoston, MA, USA; e Agence de la santé et des services sociaux de Montréal, Direction de santé publiqueMontreal, QC, Canada

**Keywords:** Influenza vaccine, mass vaccination, public health

## Abstract

**Background:**

Nineteen mass vaccination clinics were established in Montreal, Canada, as part of the 2009 influenza A/H1N1p vaccination campaign. Although approximately 50% of the population was vaccinated, there was a considerable variation in clinic performance and community vaccine coverage.

**Objective:**

To identify community- and clinic-level predictors of vaccine uptake, while accounting for the accessibility of clinics from the community of residence.

**Methods:**

All records of influenza A/H1N1p vaccinations administered in Montreal were obtained from a vaccine registry. Multivariable regression models, specifically Bayesian gravity models, were used to assess the relationship between vaccination rates and clinic accessibility, clinic-level factors, and community-level factors.

**Results:**

Relative risks compare the vaccination rates at the variable's upper quartile to the lower quartile. All else being equal, clinics in areas with high violent crime rates, high residential density, and high levels of material deprivation tended to perform poorly (adjusted relative risk [ARR]: 0·917, 95% CI [credible interval]: 0·915, 0·918; ARR: 0·663, 95% CI: 0·660, 0·666, ARR: 0·649, 95% CI: 0·645, 0·654, respectively). Even after controlling for accessibility and clinic-level predictors, communities with a greater proportion of new immigrants and families living below the poverty level tended to have lower rates (ARR: 0·936, 95% CI: 0·913, 0·959; ARR: 0·918, 95% CI: 0·893, 0·946, respectively), while communities with a higher proportion speaking English or French tended to have higher rates (ARR: 1·034, 95% CI: 1·012, 1·059).

**Conclusion:**

In planning future mass vaccination campaigns, the gravity model could be used to compare expected vaccine uptake for different clinic location strategies.

## Background

In the summer of 2009, the Canadian Public Health Agency ordered over 50 million doses of the influenza A/H1N1p vaccine, enough to administer, free of charge, one dose to each Canadian, or two doses to approximately 75% of the population. The timing of vaccine distribution to local health departments allowed for the administration of vaccines by early November.^1^ In Montreal, Canada, nineteen mass vaccination clinics (MVC) were established throughout the city with the choice of location guided by site availability and capacity. Most clinics opened their doors to the general public on November 5, 2009, 2 weeks after the start of the second wave of the pandemic in Canada.^2^ Vaccinations were delivered in priority sequence, with the goal of vaccinating at least 70% of the population. Montreal residents were free to attend any MVC in Montreal, which were open from 8 am to 8 pm, 7 days per week. By the end of the vaccination campaign, approximately 50% of Montreal's 1·8 million residents had been vaccinated. Despite the relatively high coverage rate (e.g., compared to France, 8%, and the United States, 27%),^3^,^4^ there was a considerable variation in vaccine uptake across Montreal neighborhoods.^5^

Previous studies of pandemic A/H1N1 vaccination have identified population-level determinants of vaccine coverage, for example Ref. 6, without addressing the accessibility and other characteristics of healthcare services. A simultaneous analysis of the geographic variation of population characteristics and MVC-level characteristics could elucidate predictors of regional vaccine coverage and could inform a more coordinated planning of mass vaccination campaigns. Gravity models 7 are well suited to this task as they examine the features of origin and destination that affect traffic, or flow, from origin to destination. Typically, the shorter the distance between origin and destination and the larger the mass of the origin/destination (e.g., population size/clinic capacity), the greater the “gravitational pull” or flow between the two. In this study, gravity models were used to examine the characteristics of place of residence and characteristics of MVC that were associated with flow of individuals to be vaccinated against influenza A/H1N1p. Using a simple example, we illustrate how the findings from this study could be used to inform the placement of MVC.

## Materials and methods

### Data

The pandemic A/H1N1 vaccination records were obtained from the National Public Health Institute of Québec (Institut national de santé publique du Québec). Each record contains data collected at the MVC at the time of vaccination. These include the date of vaccination, the individual's address, age, sex, and any condition (chronic disease or pregnancy) that granted the individual priority status. Although chronic diseases and pregnancy were self-reported, proof of these conditions was often requested while vaccination was restricted to priority groups. Individuals that were vaccinated at non-MVC locations (e.g., healthcare workers vaccinated at their place of work) were excluded from the analysis, that is, from both the numerators and the denominators of the vaccination rates.

Vaccination records were aggregated at the level of both census tract (CT) and MVC, as the measure of interest is the number of people from each CT that were vaccinated at each MVC. The CT unit of analysis represented a compromise between the statistical precision of vaccination rates of CT populations at each MVC and the accuracy of measurement of travel time between place of residence and MVC. Census data were suppressed or missing for 15 of 515 CTs of the Island of Montreal; these CTs were omitted from the analysis as the percentage of the total vaccinations from these areas was under 0·3%.

The research literature on the community-level determinants of vaccine uptake informed the selection of CT-level variables to include as covariates in the regression model (Description and Reference(s), Table 1). Based on the literature, the model should include a marker of material deprivation (unemployment, poverty, post-secondary education, or material deprivation index), the proportion of the population that had recently immigrated (new immigrant), the proportion of individuals that spoke either French or English (official languages), and the proportion of the population that was male. Other covariates included the proportion of the population belonging to each priority group (i.e., pregnant women; chronically ill under 65 years of age; age groups: 6 months to 4 years, 5–19 years, and 65 years and older). Data on CT population sizes and demographics were obtained from the 2006 Canadian census.^8^ The number of pregnancies and chronically ill individuals under 65 years of age were estimated using survey and demographic data, as described by Brien *et al*.^5^

**Table 1:** Variables and data sources for the Montreal census tracts in 2009

Variable	Source	Description	Median (1st quartile, 3rd quartile)
Population	Census 2006	Number of CT residents excluding individuals that were vaccinated at non-MVC locations	3140 (2142, 4261)
Material deprivation index	Census 2006	Index comprising the proportion of the population without high school diploma, employment to population ratio, and average income. Expressed as a percentile/100^5^	0·51 (0·31, 71·0)
Unemployment	Census 2006	Unemployment rate^23^,^30^	0·083 (0·062, 0·11)
Post-secondary education	Census 2006	Proportion with a post-secondary education^22^,^25^,^28^,^30^,^32^	0·68 (0·58, 0·76)
Poverty	Census 2006	Proportion of families that are living below the poverty level, that is, income <63% of the average income in their community-size, family-size strata^5^,^36^	0·22 (0·15, 0·31)
New immigrants	Census 2006	Proportion of the population that recently immigrated^5^,^29^,^34^	0·056 (0·033, 0·091)
Official languages	Census 2006	Proportion of the population (≥15 years) speaking English or French^34^,^36^	0·016 (0·0070, 0·034)
Ages 0–4	Census 2006	Proportion of the population that is 4 years old or younger^3^,^20^,^26^,^27^,^29^,^30^,^33^	0·043 (0·036, 0·050)
Ages 5–19	Census 2006	Proportion of the population that is 5–19 years old (inclusive)^3^,^20^,^26^,^27^,^29^,^30^,^33^	0·15 (0·12, 0·18)
Ages 20–64	Census 2006	Proportion of the population that is 20–64 years old (inclusive)^3^,^20^,^26^,^27^,^29^,^30^,^33^	0·64 (0·60, 0·71)
Ages 65 plus	Census 2006	Proportion of the population that is 65 years old or older^3^,^20^,^26^,^27^,^29^,^30^,^33^	0·14 (0·10, 0·18)
Chronic conditions	Estimated from CCHS* and demographic variables**	Proportion of the population under 65 years of age with a chronic condition^3^,^22^,^26^,^29^,^30^	0·134 (0·129, 0·137)
Pregnant	Estimated from CCHS and demographic variables	Proportion of the population that is pregnant^3^,^26^,^30^	0·011 (0·0094, 0·013)

* Canadian Community Health Survey.^31^

** Described by Brien *et al*.^5^

The MVC variables and their descriptions are presented in Table 2. The minimum time to drive from the CT centroid to the MVC address was estimated using the Google Directions API.^9^ In addition to the time to drive from CT to MVC, time to travel by public transit (bus and subway systems) was considered. These data were obtained from the MADITUC group (Modèle d'Analyse Désagrégée des Itinéraires de Transport Urbain Collectif). (G. Bisaillon, personal communication) Trip durations were calculated using empirically based estimates of point-to-point travel time (e.g., station-to-station time for subway-only trip), and official headways in the case of multileg trips. Time to walk to and from transit access points (bus stops or subway stations) was excluded from trip time estimates.

**Table 2:** Variables and data sources describing the areas (in 2009) in which the MVC were placed

Variable name	Source	Description	Median (1st quartile, 3rd quartile)
Violent crime rate	Service de police de la ville de Montréal	Number of violent crimes per 10 000 population	119·3 (103·6, 188·1)
Material deprivation index	Census 2006	Percentile for CT in which the MVC is placed	0·57 (0·35, 0·75)
Residential density	Census 2006	Number of dwelling units per squared kilometer of the CT in which the MVC is located (10 000 DU/km^2^*)	0·24 (0·083, 0·37)
Capacity	Institut national de santé publique du Québec	Maximum total number of vaccinations that could be administered during the vaccination campaign	84 480 (72 960, 135 800)

* Dwelling units per square kilometer.

### Statistical analysis

Gravity models are multivariable regression models that simultaneously assess the effect of characteristics of origin (place of residence) and destination (MVC) on flow from origin to destination. The outcome in the models was the number of people living in a CT that were vaccinated at a MVC. The outcome variable (*Y*_*ij*_) represents the flow from CT *i* to MVC *j*. In addition to origin and destination variables, the gravity model accounts for accessibility of the destination from the origin. In this context, accessibility of a MVC from a CT is a function of the travel time and the MVC size (i.e., vaccination capacity). Congdon^7^ introduced a modification to accessibility, called relative accessibility, because the decision to attend a MVC depends not only on its accessibility, but also on the accessibility of other MVC.

The gravity model with MVC variables and CT variables can be expressed as:


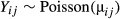



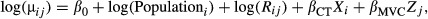



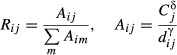


where μ_*ij*_ is the expected flow from CT *i* to MVC *j*, ***X***_***i***_ is the vector of CT variable values for CT *i*, ***Z***_***j***_ is a vector of MVC variable values for MVC *j*, *R*_*ij*_ is the relative accessibility, *A*_*ij*_ is the accessibility of MVC *j* from CT *i*, C_*j*_ is the cumulative MVC capacity (maximum total number of vaccinations that could be administered over the entire vaccination campaign), and *d*_*ij*_ is the travel time from CT *i* to MVC *j*.

We also considered hierarchical gravity models with both spatially correlated and exchangeable CT random effects to account for any unmeasured predictors of CT vaccine coverage that vary with and without spatial structure, respectively.^10^ The spatially correlated random effects were assigned a conditional autoregressive (CAR) prior.^11^ All models had, as offsets, the log-transformed CT population size and relative accessibility. All multivariable models without random effects (i.e., pooled regressions) adjusted for the proportion of the population belonging to each priority group and the proportion of males in the CT. Due to the complexity of the hierarchical models, for example the spatially correlated random effects, all models were implemented in a Bayesian framework using Markov Chain Monte Carlo techniques. See Appendix 1 for details of the statistical analysis.

### 

#### Application of gravity model to assess alternative geographic distributions of MVC

Using the gravity model as a predictive model, we determined whether an alternative location for one of the MVC would likely improve vaccine uptake. The new MVC had the same total capacity but was more centrally located in a community with a lower residential density, less material deprivation, and a lower violent crime rate. As inputs in the gravity model to predict the number of vaccinations at this new clinic, we used the drive times from all communities to the clinic, as well as the area's residential density, violent crime rate, and material deprivation.

#### Implementation

A Bayesian approach was used with minimally informative normal prior distributions centered at the null, that is, N(0, 1000), for all regression coefficients except the intercept which was assigned a non-informative prior. For the hierarchical models, the inverse variances from the random effects prior distributions were assigned gamma(0·1, 0·0001) priors. All CT and MVC variables were centered to improve convergence of the Markov Chain Monte Carlo (MCMC) sequences. Three chains were produced, each with 15 000 iterations for the multivariable (pooled) regression models and up to 100 000 iterations for the hierarchical models. The first half of the MCMC chains were discarded as burn-in, keeping every 5th estimate in the chain for posterior summaries of the regression parameters of the model. The potential scale reduction factor was used to assess convergence.^12^,^13^ The deviance information criterion (DIC) guided model selection. Many of the candidate CT variables were highly correlated (correlation > 0·5), 14,15 which could potentially introduce multicollinearity and make the interpretation of the results challenging. Avoiding multicollinearity was prioritized in constructing candidate models, for example, no more than one variable representing material deprivation was included in a model. All models were run in WinBUGS 1.4 16 and R 2.13.2 17 software using the R2Winbugs package 18 to link WinBUGS and r.

#### Sensitivity analysis

Three of the 19 MVC were open for <12 days, while most others were open for the full 44 days of the mass vaccination campaign. Although the MVC capacity variable in the relative accessibility term would reflect this, the time period during which these MVC were open could be qualitatively different than the time period during which they were closed. Analyses were carried out both with and without these MVC. In addition, sensitivity analyses were conducted on the mode of travel, carrying out analyses using, first, time to drive from CT to MVC and then using time to travel by public transit. We also assessed sensitivity of the results to the choice of prior distributions by re-running the analyses with alternative priors (Table A3, footnotes).

## Results

**Figure 1:**
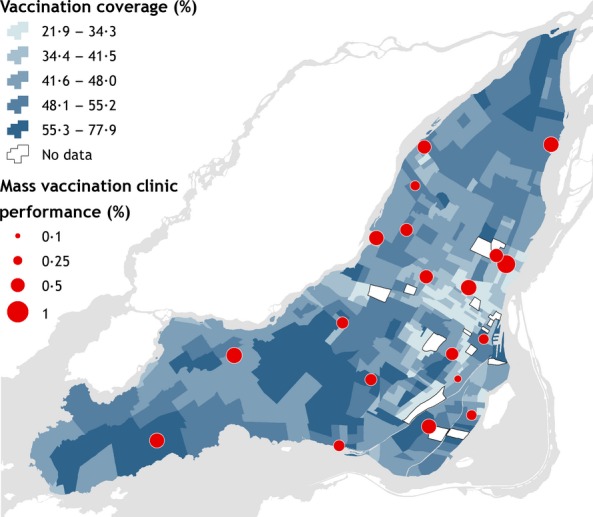
Geographic position of MVC, MVC performance, and CT vaccination coverage.

**Figure 2:**
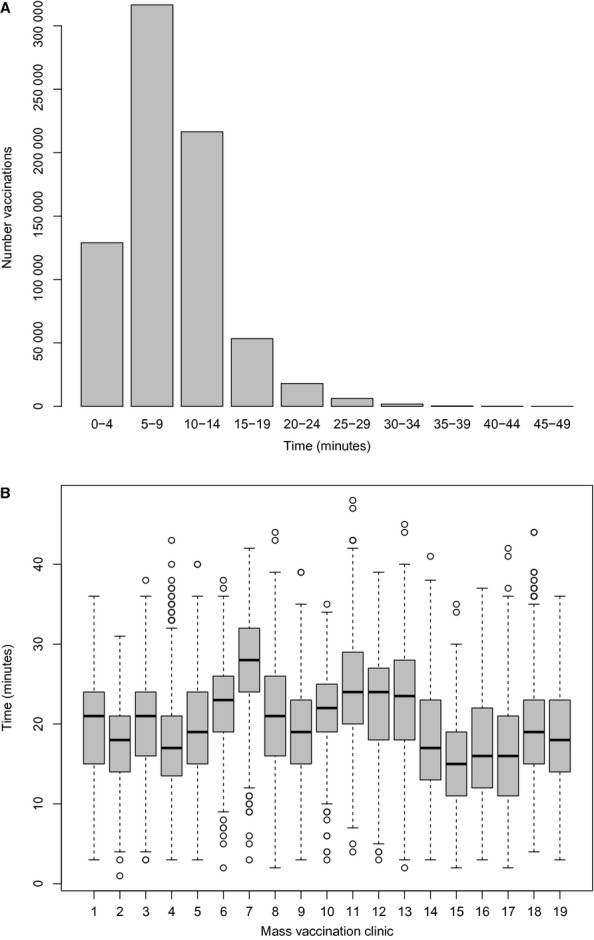
Observed travel times by car (A). Distribution of travel times from CT to MVC (B).

There were 741 237 vaccinations administered at Montreal MVC between November 5, and December 18, 2009. Overall MVC performance (measured by the total number of vaccinations at a MVC divided by the total MVC capacity) varied from 0·17 to 0·75 with an average performance of 0·44. CT vaccine coverage ranged from 0·22 to 0·78 with an average coverage of 0·44 (Figure 1). Approximately 90% of the observed driving times to MVC from place of residence were <15 minutes (Figure 2A), although the median of the distribution of driving times for all CT/MVC pairs was 20 minutes (Figure 2B).

Tables of model fit and results of the sensitivity analyses are presented in Tables A1–A4. The results of the “best” gravity model, according to the DIC (Table A1), are shown in Table 3. This model was based on time to drive from CT to MVC and included CT-level exchangeable and spatially correlated random effects, the percentage of the CT population speaking English/French, the proportion living below the poverty level, the proportion that are new immigrants, the proportion 4 years old or younger, the proportion of males, and all the MVC covariates, that is, the violent crime rate, material deprivation score, and residential density. When added to the models with CT covariates new immigrants, poverty, and official languages, the MVC predictors substantially decreased the DIC, demonstrating the predictive power of the MVC variables (multivariable pooled regression model 588 464 versus 557 421; hierarchical model 580 634 versus 528 563). We were only able to adjust for one priority group (proportion ≤ 4 years old) in the hierarchical model, because more complex hierarchical models did not converge. The adjusted relative risks (ARR) that are presented compare the vaccination rate calculated at the upper quartile to the vaccination rate calculated at the lower quartile of the CT/MVC variables. Quartiles of the CT/MVC variable distributions are shown in Tables 1 and 2. The positive regression coefficients for driving time (4·13, 95% credible interval [CI]: 4·12, 4·14) and capacity (1·098, 95% CI: 1·092, 1·104) suggest that, all else being equal, individuals tended to use MVC that were closer to home and MVC that had a greater vaccination capacity (Table 3). After adjusting for other measured and unmeasured CT-level variables, MVC variables, and relative accessibility, CTs with higher proportions of residents living below the poverty level and CTs with a greater proportion of new immigrants tended to have lower vaccine uptake (ARR: 0·918, 95% CI: 0·893, 0·946; ARR: 0·936, 95% CI: 0·913, 0·959, respectively). CTs with a greater proportion speaking an official language tended to have higher vaccination rates (ARR: 1·034, 95% CI: 1·012, 1·059). For the MVC variables, all else being equal, fewer vaccinations took place at clinics located in areas with high residential density and high violent crime rates (ARR: 0·663, 95% CI: 0·660, 0·666; ARR: 0·917, 95% CI: 0·915, 0·918, respectively). There was also reduced flow to clinics placed in areas with high material deprivation (ARR: 0·649, 95% CI: 0·645, 0·654).

**Table 3:** Regression coefficients and relative risks from the analysis of flows from Montreal CTs to MVC during the 2009 influenza pandemic mass vaccination campaign

Variable	Regression coefficient*^,^ **	95% Credible interval
Capacity (exponent)	1·098	1·092, 1·104
Drive time (exponent)	4·13	4·12, 4·14

CT variable	Relative risk*^,^**	95% Credible interval
New immigrant	0·936	0·913, 0·959
Poverty	0·918	0·893, 0·946
Official languages	1·034	1·012, 1·059

MVC variable	Relative risk*^,^**	95% Credible interval
Material deprivation	0·649	0·645, 0·654
Violent crime rate	0·917	0·915, 0·918
Residential density	0·663	0·660, 0·666

* Hierarchical model adjusting for driving time, clinic capacity, proportion age ≤4 years, proportion of males and unmeasured CT variables (CT random effects).

** Relative risks compare vaccination rates at the upper to vaccination rates at the lower quartile of the CT/MVC variable (Tables 1–2).

Replacing a clinic located in an area with high material deprivation, residential density, and violent crime rate by a clinic located in an area with less material deprivation, a lower residential density and a lower violent crime rate resulted in a greater predicted clinic performance (54 938 predicted vaccinations in the new clinic, compared to 20 730). The material deprivation score, residential density, and violent crime rates for the new compared to the original area were 51·0 versus 80·1, 1211 versus 3754 per 10 000 DU/km^2^, and 61·1 versus 205·8 per 10 000, respectively.

After omitting the three MVC that were open for <2 weeks, there was little change in the regression coefficients (Table A2). Similarly, comparing the results from the model with alternative priors, there was little change in the regression coefficients (Table A3). According to the DIC, models with time to travel by car fit the data better than the same model with time to travel by public transit (Table A1). There were differences between the two models, the most notable being the association with travel time (exponent for drive time: 4·13, 95% CI: 4·12, 4·14; exponent for time by public transit 0·0242, 95% CI: 0·0191, 0·0296) (Table A4).

## Discussion

Our study findings suggest that clinic accessibility, community-level factors, and clinic-level factors were predictors of vaccine uptake. Individuals tended to get vaccinated at clinics that were closer to home and had a greater capacity. Community of residence characteristics were associated with vaccine uptake. All else being equal, community populations that had a high proportion of new immigrants and a high proportion of individuals living below the poverty line tended to have lower vaccine uptake; compared to the lower quartile of the distribution of proportion of new immigrants and proportion living below the poverty line, those at the upper quartile had average decreases in vaccination rates of 6% and 8%, respectively. Speaking an official language, that is, either English or French, was associated with a 3% higher vaccine uptake. Adding clinic-level factors to the models with clinic accessibility and community-level covariates greatly improved the models' predictive power. After accounting for clinic accessibility and community-level variables, there were decreased vaccination rates associated with clinics in areas with higher residential density, higher violent crime rates, and higher material deprivation. Compared to clinics located in areas with residential density, violent crime rate, and material deprivation at the lower quartile, those at the upper quartile had at least a 33%, 8% and 35% reduction in vaccination rates. The gravity model that accounts for clinic accessibility and both community- and clinic-level factors could be used to identify alternative geographic positions of the clinics that have higher predicted vaccine coverage.

Influenza A/H1N1p vaccine coverage not only varied considerably between countries but there is also evidence of regional variation at a smaller scale.^3^,^19^–^21^ Previous studies have related recent immigration, ethnicity, level of education, occupation, income, age, gender, and material deprivation with vaccine uptake.^3^,^5^,^6^,^20^,^22^–^33^ Most studies found increased uptake with higher education^22^,^25^,^28^,^30^,^32^ and risk group status,^3^,^20^,^25^,^26^,^28^–^30^ but there was little consistency in findings between studies with respect to age and sex.^3^,^20^,^23^,^26^,^27^,^29^–^31^,^33^ For example, some studies found that older populations more likely to be vaccinated, 23,26,27,29,31,33 while others found higher uptake among children.^20^,^22^,^30^

All else being equal, lower vaccine uptake was more typical of areas with a higher proportion of new immigrants and this is consistent with the literature regarding healthcare utilization/vaccine uptake among recent immigrants in Canada.^5^,^34^,^35^ Interestingly, the association persisted even after adjusting for the percentage of the CT population speaking an official language, which underlines the complexity of the relationship between ethnicity/immigrant status and healthcare utilization. Deri 36 found that the spread of information regarding healthcare issues, such as the opening of vaccination clinics, can be facilitated in communities with a high concentration of an ethnic group, but if ethnic norms do not support healthcare utilization/vaccination, this will not necessarily result in greater healthcare utilization/vaccination uptake. On the other hand, there tends to be greater healthcare utilization in ethnic communities when there are a greater number of physicians from that ethnicity practicing medicine in the community.^36^ Thus, in promoting vaccination in communities, sensitivity to the population's ethnic composition and spoken languages, for example by involving ethnic healthcare personnel, may encourage vaccination uptake.

Time to drive from home to the clinic was an important factor in deciding where to be vaccinated. The median driving time for all CT/MVC pairs was 20 minutes and the longest trip was about 50 minutes, but approximately 90% of the trips took under 15 minutes. The models with accessibility based on time to travel by public transit did not fit the data as well as time to travel by car. However, our measures of travel time by public transit did not include time to walk to and from transit access points. Nevertheless, MVC in areas with high residential density did not perform well in general, including two of the MVC that were placed in very close proximity to subway stations. These suggest that drive time and access to parking at the MVC should be considered when planning MVC placement.

The impact of geographic accessibility to healthcare services has been examined in previous studies although different aspects of accessibility were studied. Fu *et al*.^37^ reported that young children in Washington, DC, with greater spatial accessibility were more likely to be up-to-date with respect to vaccinations. Spatial accessibility in their study was a measure of population-to-provider ratio for each residence of the study region. Baumgardner *et al*.^38^ conducted a chart review of 2- to 5-year-old patients from two clinics in Milwaukee, Wisconsin, to determine whether distance from home to clinic was related to the level of completion of vaccinations. They did not find evidence of an association between distance and level of completion of vaccinations.

Strengths of this study include the quality of the data, which were compiled from a vaccine registry rather than data derived from surveys. The analysis was based on a fine geographic partition, into CTs, which provides reassurance of within-community homogeneity in exposures. Even so, the study was limited by the aggregated nature of our data. Causal relationships at the individual level cannot necessarily be inferred from these findings. However, public health interventions are generally directed at communities [e.g., telephone or text messaging reminders to community residents with low vaccine uptake^39^], thus supporting the study of these relationships at the level of community and clinic rather than the individual. Nevertheless, we can only make inferences about the factors that promoted vaccination, with caution.

The findings from this study suggest that poverty, new immigrant status, and not speaking an official language were associated with decreased vaccine uptake. For equally accessible clinics, those located in regions with high residential density, material deprivation, and violent crime rates did not perform as well. Distance was also an important factor as most trips took under 15 minutes by car. The gravity models we developed could in similar circumstances be used to help identify MVC placements that would result in greater vaccine coverage. In addition, preparations for future mass vaccination campaigns should also consider different ways in which important information can be more effectively transmitted to new immigrants, populations that do not speak English or French, and those living in poverty.^40^ Possible ways to reach these vulnerable populations include advertising in freely accessible local newspapers, advertising in ethnic newspapers, and during ethnic television programs, advertising in local community centers and holding information sessions using personnel from the appropriate ethnic group.

## Appendix 1

Gravity models are used to analyze the flow of people, commodities, information, etc., from an origin to a destination. The origin and destination are characterized by their mass (e.g., population size) and other attributes (e.g., urban versus rural). Distance between origin and destination also contributes to the “gravitational pull” or flow between origin and destination. In general, decreased masses and increased distances are associated with diminished flow. Gravity models are used to examine the role of supply, demand, distance, and origin/destination characteristics on flow from origin to destination.

**Table A1:** Deviance information criterion

Model	Covariates	DIC
Multivariable regression*	None	590 361
	Official languages	590 195
	Post-secondary education	590 193
	MVC violent crime rate	589 869
	CT material deprivation index	589 796
	New immigrants	589 389
	Unemployment	589 065
	Poverty	588 637
	New immigrants + poverty	588 499
	New immigrants + poverty + official languages	588 464
	Ratio of MVC to CT of residence material deprivation	587 898
	MVC material deprivation index	580 674
	MVC residential density	571 158
	New immigrants + poverty + official languages + MVC material deprivation + MVC residential density + MVC violent crime rate	557 421
Hierarchical**	None	580 639
	New immigrants + poverty + official languages	580 634
	New immigrants + poverty + official languages + MVC material deprivation + MVC residential density + MVC violent crime rate	528 563
Hierarchical *** (public transit)	New immigrants + poverty + official languages + MVC material deprivation + MVC residential density + MVC violent crime rate	2 733 460

* Pooled regression adjusting for driving time, clinic capacity, proportion of the population belonging to each priority group, and proportion of males.

** Adjusting for driving time, clinic capacity, proportion age ≤4 years, proportion of males and unmeasured CT variables (CT random effects).

*** Adjusting for time by public transit, clinic capacity, proportion age ≤4 years, proportion of males and unmeasured CT variables (CT random effects).

Flow from origin i to destination *j*, *Y*_*ij*_ is assumed to follow a Poisson distribution with mean μ_*ij*_ and

where *k* is a constant, is a distance decay factor, *D*_*i*_ represents the demand at origin *i*, and *C*_*j*_ signifies the supply at destination *j*. Accessibility of destination *j* from origin *i* can be expressed as  giving an alternative expression for mean flow from origin *i* to destination *j*, based on accessibility,

Because the preference for one destination depends on the accessibility from the origin to all other destinations, Congdon (2001) introduced relative accessibility, , and the mean flow is re-expressed as

Taking the logarithm of both sides and adding origin- and destination-specific covariates give the log-linear model:

where **X**_***i***_ and **Z**_***i***_ are the vectors of covariate values for origin *i* and destination *j*, respectively, and ***β*_origin_** and *β*_**destination**_ are the regression coefficients for the origin and destination covariates, respectively. In the Bayesian approach presented by Congdon,^7^ minimally informative normal prior distributions are assigned to the regression coefficients. Taking population size as the demand, the Bayesian gravity model formulation is:

Alternatively, rather than including log (population_*i*_) and log (*R*_*ij*_) as offsets, they may be entered in the model as covariates. To account for unmeasured risk factors that vary with and without spatial structure, spatially correlated random effects and exchangeable random effects, respectively, can be added to the model.^10^ We can assume that the exchangeable random effects follow a normal distribution with zero mean. Spatially correlated random effects can follow a CAR prior, which assumes that neighboring areas have similar event rates. The model with exchangeable and spatially correlated random effects is:

*δ*_*i*_ is the set of neighbors of area *i*, for example areas sharing a border with area *i*, and *n*_*i*_ is the number of neighbors. Typically, the inverses of the variances  and  are assigned gamma priors.

**Table A2:** Regression coefficients and relative risks from the analysis excluding three MVC, open for short durations

Variable	16 MVC		19 MVC	
	
	Regression coefficient*	95% Credible interval	Regression coefficient*	95% Credible interval
Capacity (exponent)	1·426	1·415,1·438	1·098	1·092, 1·104
Drive time (exponent)	4·362	4·355, 4·371	4·13	4·12, 4·14

CT variable	Relative risk*^,^**	95% Credible interval	Relative risk*^,^**	95% Credible interval
New immigrant	0·936	0·910, 0·964	0·936	0·913, 0·959
Poverty	0·912	0·883, 0·946	0·918	0·893, 0·946
Official languages	1·050	1·021, 1·081	1·034	1·012, 1·059

MVC variable	Relative risk*^,^**	95% Credible interval	Relative risk*^,^**	95% Credible interval
Material deprivation	0·639	0·634, 0·643	0·649	0·645, 0·654
Violent crime rate	0·907	0·906, 0·908	0·917	0·915, 0·918
Residential density	0·606	0·603, 0·609	0·663	0·660, 0·666

* Hierarchical model adjusting for driving time, clinic capacity, proportion age ≤4 years, proportion of males and unmeasured CT variables (CT random effects).

** Relative risks compare vaccination rates at the upper to vaccination rates at the lower quartile of the CT/MVC variable (Tables 1–2).

**Table A3:** Regression coefficient and relative risks (hierarchical model) from the sensitivity analysis of prior distributions

Variable	Main priors*		Alternative priors**	
	
	Regression coefficient***	95% Credible interval	Regression coefficient***	95% Credible interval
Capacity (exponent)	1·098	1·09, 1·10	1·098	1·09, 1·11
Travel time (exponent)	4·13	4·12, 4·14	4·13	4·12, 4·14

CT variable	Relative risk***^,^ †	95% Credible interval	Relative risk***^,^ †	95% Credible interval
New immigrant	0·936	0·913, 0·959	0·938	0·915, 0·961
Poverty	0·918	0·893, 0·946	0·917	0·891, 0·943
Official languages	1·034	1·012, 1·059	1·035	1·012, 1·058

MVC variable	Relative risk***^,^ †	95% Credible interval	Relative risk***^,^ †	95% Credible interval
Material deprivation	0·649	0·645, 0·654	0·649	0·645, 0·654
Violent crime rate	0·917	0·915, 0·918	0·917	0·915, 0·918
Residential density	0·663	0·660, 0·666	0·663	0·660, 0·666

* Main priors: intercept: non-informative; regression coefficients N(0, 1000); inverse variances gamma(0·1, 0·0001).

** Alternative priors: intercept: non-informative; regression coefficients N(0, 10); inverse variances gamma(0·01, 0·0001).

*** Hierarchical model adjusting for driving time, clinic capacity, proportion age ≤4 years, proportion of males and unmeasured CT variables (CT random effects).

† Relative risks compare vaccination rates at the upper to vaccination rates at the lower quartile of the CT/MVC variable (Tables 1–2).

**Table A4:** Regression coefficient and relative risks (hierarchical model) from the sensitivity analysis of prior distributions

Variable	Main priors*		Alternative priors**	
	
	Regression coefficient***	95% Credible interval	Regression coefficient***	95% Credible interval
Capacity (exponent)	1·098	1·09, 1·10	1·098	1·09, 1·11
Travel time (exponent)	4·13	4·12, 4·14	4·13	4·12, 4·14

CT variable	Relative risk***^,^ †	95% Credible interval	Relative risk***^,^ †	95% Credible interval
New immigrant	0·936	0·913, 0·959	0·938	0·915, 0·961
Poverty	0·918	0·893, 0·946	0·917	0·891, 0·943
Official languages	1·034	1·012, 1·059	1·035	1·012, 1·058

MVC variable	Relative risk***^,^ †	95% Credible Interval	Relative risk***^,^ †	95% Credible interval
Material deprivation	0·649	0·645, 0·654	0·649	0·645, 0·654
Violent crime rate	0·917	0·915, 0·918	0·917	0·915, 0·918
Residential density	0·663	0·660, 0·666	0·663	0·660, 0·666

* Main priors: intercept: non-informative; regression coefficients N(0, 1000); inverse variances gamma(0·1, 0·0001).

** Alternative priors: intercept: non-informative; regression coefficients N(0, 10); inverse variances gamma(0·01, 0·0001).

*** Hierarchical model adjusting for driving time, clinic capacity, proportion age ≤4 years, proportion of males and unmeasured CT variables (CT random effects).

† Relative risk compare vaccination rates at the upper to vaccination rates at the lower quartile of the CT/MVC variable (Tables 1–2).
